# Association of serum Nrf2 protein levels with disease activity and renal impairment in lupus nephritis

**DOI:** 10.3389/fimmu.2024.1304167

**Published:** 2024-01-18

**Authors:** Jicui Li, Qiaoyan Guo, Xianping Wei, Yuexin Zhu, Manyu Luo, Ping Luo

**Affiliations:** ^1^Department of Nephrology, The Second Hospital of Jilin University, Changchun, China; ^2^Department of Clinical Research, The Second Hospital of Jilin University, Changchun, China

**Keywords:** lupus nephritis, oxidative stress, nuclear factor erythroid 2-related factor 2, systemic lupus erythematosus, disease activity index, estimated glomerular filtration rate

## Abstract

**Introduction:**

We aimed to investigate the relationship between nuclear factor erythroid 2-related factor 2 (Nrf2) protein expression levels, lupus nephritis (LN) disease activity, and the degree of renal injury (based on the estimated glomerular filtration rate [eGFR]) in patients with LN.

**Methods:**

We selected 40 healthy control participants and 102 patients with LN who were treated in the Second Hospital of Jilin University, China, for inclusion in this study. Patients with LN were classified into LN with high-eGFR and LN with low-eGFR groups. Nrf2 protein levels were measured in the serum and renal tissues of the participants in both groups to assess the correlation between Nrf2 protein levels and different LN disease states.

**Results:**

There was a significantly positive correlation between serum Nrf2 protein levels, the degree of renal injury, and systemic lupus erythematosus disease activity index (SLEDAI) scores in patients with LN. Nrf2 protein levels were higher in the LN with high-eGFR group than in the healthy control and LN with low-eGFR groups. In follow-up patients in the LN high eGFR group, Nrf2 protein levels decreased significantly after remission of disease activity.

**Conclusion:**

Nrf2 protein expression has a dual role in patients with LN. Nrf2 protein levels not only correlate with disease activity in patients with LN, but also with the degree of kidney injury. Before implementing targeted therapy for Nrf2, evaluating both Nrf2 protein expression and the disease state in patients with LN is necessary to better identify and place each patient in an appropriate patient group.

## 1 Introduction

Lupus nephritis (LN) is an autoimmune inflammatory glomerular disease caused by systemic lupus erythematosus (SLE) and involves the kidneys. Approximately 10–20% of patients with LN develop end-stage renal disease (ESRD) within 10 years (1). Excessive production of reactive oxygen species (ROS) in patients with SLE in response to noxious stimuli may affect the antioxidant capacity of the body (2, 3). Nuclear factor erythroid 2-related factor 2 (Nrf2) is a central regulator of oxidative stress and inflammation (4); a growing number of reports in the literature support a role of Nrf2 in LN. Nrf2 is involved in anti-apoptotic, anti-inflammatory, antioxidant, epithelial-mesenchymal transition (EMT) (5), and mesenchymal fibrosis processes, all of which play important roles in the progression of LN (4, 6). Nrf2-mediated oxidative stress pathways are shared among LN, diabetic nephropathy, IgA nephropathy, and other disorders, indicating that targeting Nrf2 expression may be critical to successful LN therapy (7). Studies on Nrf2 and Nrf2 inducers in LN have focused on animal models in which the Nrf2 pathway is down-regulated in renal cells. Furthermore, Nrf2 inducers have been shown to have anti-inflammatory, antioxidant, and protective effects against renal injury in LN mice (4). Currently, clinical studies on Nrf2 have focused on advanced kidney disease, with reports indicating that Nrf2 expression correlated with the degree of renal failure and comorbidities (8, 9) and Nrf2 expression varied among different types of immune cells and tissues (4). Meanwhile, regarding clinical trials, it was noted that the Nrf2 activator bardoxolone methyl did not reduce the risk of death due to ESRD in patients with type 2 diabetes and stage 4 chronic kidney disease. It led to a higher rate of cardiovascular events compared to the placebo group, leading to the termination of the trial (10), as this Nrf2 activator resulted in an increased albuminuria (10, 11). This necessitates the exploration of a uniform assay for Nrf2 protein levels and clinical evaluation of targeted Nrf2 therapy for renal disease (9). This makes it challenging to assess Nrf2 levels and administer interventions targeting Nrf2 in human LN cases. Therefore, understanding the endogenous state of Nrf2 in LN is a key to administering therapy targeting Nrf2 and would help select suitable LN patient populations for such intervention as well as promote the use of different classes of Nrf2 therapeutics in patients with LN.

## 2 Materials and methods

### 2.1 Study samples

We recruited 40 healthy controls from the Physical Examination Center and 102 patients with a definitive diagnosis of LN who underwent renal puncture biopsy at the Second Hospital of Jilin University, China for inclusion in this study (**Supplementary Table 1**). Patients with infection-related diseases, malignant tumors, severe hypertension, severe hepatic insufficiency, combination of other autoimmune diseases, and incomplete clinicopathological data were excluded. Immunohistochemical (IHC) controls (n=6) were taken from normal paracancerous renal tissues resected from patients with cancer. The included patients were followed up for a period of 6-12 months, and serum Nrf2 protein levels were measured again when the patients’ disease activity had resolved significantly. The degree of renal injury was determined according to the estimated glomerular filtration rate (eGFR) category. The patients with LN were divided into LN with high-eGFR (eGFR≥90 ml/min/1.73 m^2^) and LN with low-eGFR (eGFR<90 ml/min/1.73 m^2^) groups stratified by eGFR levels (12, 13). Investigations were conducted in accordance with the Declaration of Helsinki. Our study protocol was reviewed and approved by the Human Investigation Committee of the Second Hospital of Jilin University (Approval Number: 2023112). A written informed consent was obtained from each participant.

### 2.2 Research methods

(1) Data collection: We collected blood from patients with LN in the morning of the day of renal biopsy. We used gel-separator serum tubes and attempted to centrifuge the samples at 4°C within half an hour after sample collection. If the samples could not be processed within half an hour, we put the samples in the refrigerator to settle and processed the samples within 2 hours. Thereafter, we stored the split samples in a refrigerator at -80°C. And we performed multiple enzyme-linked immunosorbent assays (ELISA) via a kit (Nanjing, China) using serum from the patients and controls. The targets of the assay were Nrf2 (ml060303V), TNF-α (ml064303V), and NF-κB p65 (ml057460V). We retrospectively analyzed physical examination data such as sex, age, body mass index (BMI), and blood pressure. Moreover, data on albumin (Alb), fasting plasma glucose (FPG), serum creatinine (Scr), blood urea nitrogen (BUN), uric acid (UA), eGFR, triglycerides (TG), total cholesterol (TC), and laboratory data such as 24-hour urine protein and the SLEDAI score (14) were also retrospectively analyzed. The 24-hour proteinuria collection method: Generally, the process begins at 8 a.m., The patient empties urine at 8 a.m., and thereafter, all the urine is collected in a fixed container until 8 a.m. the next day, when the patient empties all the urine from the bladder into the container.

(2) Renal pathology: All renal samples from the patients with LN and healthy controls were confirmed by examination (including immunofluorescence, light microscopy and electron microscopy) by two experienced renal pathologists. Semi-quantitative scoring was used according to the International Society of Nephrology/Renal Pathology (ISN/RPS) 2003 revised staging criteria for LN and the ISN/RPS 2018 revised Activity Index (AI) and Chronicity Index (CI) (15). Using the ISN/RPS definitions, the patients with LN were classified into: class I, minimal mesangial; class II, mesangial proliferative; class III, focal; class IV, diffuse segmental or global; class V, membranous; and class VI, advanced sclerosing cases (16).

### 2.3 Immunohistochemical staining

Formalin-fixed paraffin-embedded kidney tissue sections were blocked by bovine serum albumin (3%) after dewaxing, rehydration, and heat-induced epitope repair, and stained with anti-Nrf2 (l:50; Abcam), anti-NF-κB p65 (l:50; Cell Signaling), and anti-TNF-α (1:50; Abcam) antibodies. Quantification was performed using the Image-Pro Plus software V.6.0 (Media Cybernetics, Bethesda, MD) to assess IHC staining intensity (Integrated optical density per unit area).

### 2.4 Statistical analysis

SPSS 25.0 software (IBM Corp., Armonk, NY, USA) was used for statistical analysis. Continuous data are presented as mean ± SD or median and interquartile range (IQR), as appropriate. Categorical data are described by absolute frequencies and percentages. Continuous clinical data were compared using Student’s t-test or the Mann–Whitney U test as appropriate. Bivariate correlation analysis using Pearson correlation analysis was employed to assess Nrf2 protein expression levels. Binary logistic regression was used to adjust for age and proteinuria to determine the difference in Nrf2 levels between the two groups of LN. In addition, a linear mixed model was used to identify the changes in Nrf2 protein in patients with LN achieving remission from disease activity.

## 3 Results

### 3.1 General data of the control group and LN patients

A total of 40 healthy controls were included in this study, comprising 32 women and 8 men with a mean age of 38.13 ± 11.67 years. Among 102 patients with LN, 87 were women and 15 were men with a mean age of 38.47 ± 12.87 years. Differences between the two groups were observed in renal function such as proteinuria, serum creatinine, eGFR. The patient distribution in terms of pathologic types of LN (types II/III/IV/V) was 7 (6.86%), 23 (22.55%), 51 (50.00%), and 21 (20.59%) patients, respectively. Of note, patients with type I and VI LN were not included in the present study, and the pathologic activity scores (mean AI and CI values) for these two LN types were 7.85 ± 3.40 and 3.41 ± 1.99, respectively. The detailed general data are listed in **Table 1**.

**Table 1:** Basic characteristics of participants of the study.

Parameters	Control group(n=40)	LN patients(n=102)	*P*
Age (years)	38.13 ± 11.67	38.47 ± 12.87	0.883
BMI (kg/m^2^)	23.75 ± 1.91	23.12 ± 3.77	0.193
Sex, female/male	32/8	87/15	0.441
Hypertension(n,%)	0	46,45.10%	<0.001*
Proteinuria (g/24 h)	NA	2.52(0.97,4.67)	–
Scr (μmol/L)	55.00(51.25,61.75)	75.50(57.00,120.00)	<0.001*
BUN (mmol/L)	3.99(3.55,5.04)	8.03(5.10,12.76)	<0.001*
eGFR (mL/min/1.73 m^2^)	115.49 ± 9.12	80.38 ± 38.75	<0.001*
FBG(mmol/L)	4.87(4.57,5.16)	5.21(4.70,5.85)	0.005*
CRP (mg/L)	NA	3.30(1.76,7.03)	–
Serum albumin (g/L)	44.16 ± 2.72	28.57 ± 6.30	<0.001*
TC (mmol/L)	4.37(3.99,4.79)	4.71(3.63,6.40)	0.092
TG (mmol/L)	0.90(0.78,1.12)	2.14(1.53,2.94)	<0.001*
Pathological type, II/III/IV/V	NA	7/23/51/21	–
AI	NA	7.85 ± 3.40	–
CI	NA	3.41 ± 1.99	-
Anti-dsDNA positivity (n,%)	NA	68(66.67%)	-
ANA positivity (n,%)	NA	100(98.04%)	-

AI, activity index; BMI, body mass index; BUN, blood urea nitrogen; CI, chronicity index; CRP, C-reactive protein; eGFR, estimated glomerular filtration rate; FBG, fasting blood glucose; LN, lupus nephritis; Scr, serum creatinine; TC, total cholesterol; TG, triglycerides. NA: not available. ∗*p* < 0.05.

### 3.2 Bivariate correlation analysis for Nrf2

As reported in the literature, Nrf2 protein level correlated with eGFR (17), proteinuria (18), diabetes mellitus (19), C-reactive protein (CRP) (20), and SLEDAI score (21) in renal diseases. In the present study in patients with LN, Nrf2 protein level positively correlated with eGFR and the SLEDAI score. The detailed general data are listed in **Table 2**.

**Table 2:** Bivariate correlation analysis of Nrf2 protein levels.

Parameters	r	P
eGFR(ml/min/1.73m^2^)	0.433	<0.001*
Proteinuria (g/24 h)	0.085	0.419
FBG (mmol/L)	0.042	0.688
CRP (mg/L)	0.037	0.754
SLEDAI	0.320	<0.001*
Complement C3(mg/dL)	-0.149	0.145
Anti-dsDNA positivity	0.188	0.059

CRP, C-reactive protein; eGFR, estimated glomerular filtration rate; FBG, fasting blood glucose; SLEDAI, systemic lupus erythematosus disease activity index. ∗*p* < 0.05.

### 3.3 Relationship between eGFR and Nrf2

Based on these results, we concluded that eGFR positively correlated with Nrf2. Therefore, we compared the clinical and biochemical characteristics between the LN with high-eGFR group (eGFR≥90 mL/min/1.73 m^2^) and the LN with low-eGFR group (eGFR<90 mL/min/1.73 m^2^) among patients with LN. The data showed differences in renal function: the eGFR value in the LN with low-eGFR group was significantly lower than that in the LN with high-eGFR group. However, the variables of age, proteinuria, serum creatinine, urea nitrogen, and FBG in the LN with low-eGFR group were significantly higher than that in the LN with high-eGFR group. However, there was no significant difference in the SLEDAI score, AI, and CI values between the two groups of patients with LN. The detailed general data are listed in **Table 3**.

**Table 3:** General characteristics of LN with high-eGFR and low-eGFR groups.

Parameters	LN with high-eGFR(n=56)	LN with low-eGFR(n=46)	*P*
Age (years)	36.07 ± 11.63	41.39 ± 13.80	0.037*
BMI (kg/m^2^)	23.12 ± 3.88	23.11 ± 3.69	0.985
Sex, female/male	45/11	42/4	0.120
Hypertension (n,%)	22(39.29%)	23(50.00%)	0.278
Proteinuria (g/24 h)	1.81(0.66,4.53)	3.35(1.77, 5.82)	0.005^*^
Scr (*μ*mol/L)	59.00(48.50,71.00)	105.50(88.25,169.00)	<0.001^*^
BUN (mmol/L)	5.74(4.23,9.64)	9.02(7.74,16.62)	<0.001^*^
FBG, (mmol/L)	4.96(4.49,5.41)	5.66(5.08,6.20)	<0.001^*^
CRP (mg/L)	3.30(1.10,5.79)	4.58(3.30,9.18)	0.062
Serum albumin (g/L)	29.59 ± 6.41	27.21 ± 5.95	0.064
TC (mmol/L)	4.40(3.49,5.77)	5.19(4.44,6.75)	0.128
TG (mmol/L)	2.04(1.47,2.52)	2.40(1.55,3.33)	0.057
Pathological type, II/III/IV/V	6/12/27/11	1/11/24/10	-
SLEDAI	13.34 ± 4.53	13.04 ± 3.40	0.710
AI	7.48 ± 3.74	8.27 ± 2.89	0.250
CI	3.36 ± 2.13	3.48 ± 1.82	0.766
Anti-dsDNA positivity (n,%)	39(69.64%)	29(63.04%)	0.482
ANA positivity (n,%)	55(98.21%)	45(97.83%)	0.888

AI, activity index; BMI, body mass index; BUN, blood urea nitrogen; CI, chronicity index; CRP, C-reactive protein; eGFR, estimated glomerular filtration rate; FBG, fasting blood glucose; LN, lupus nephritis; Scr, serum creatinine; SLEDAI, systemic lupus erythematosus disease activity index; TC, total cholesterol; TG, triglycerides. ∗p < 0.05.

### 3.4 Differences in Nrf2, TNF-α, and NF-κB p65 protein levels

**Figure 1:**
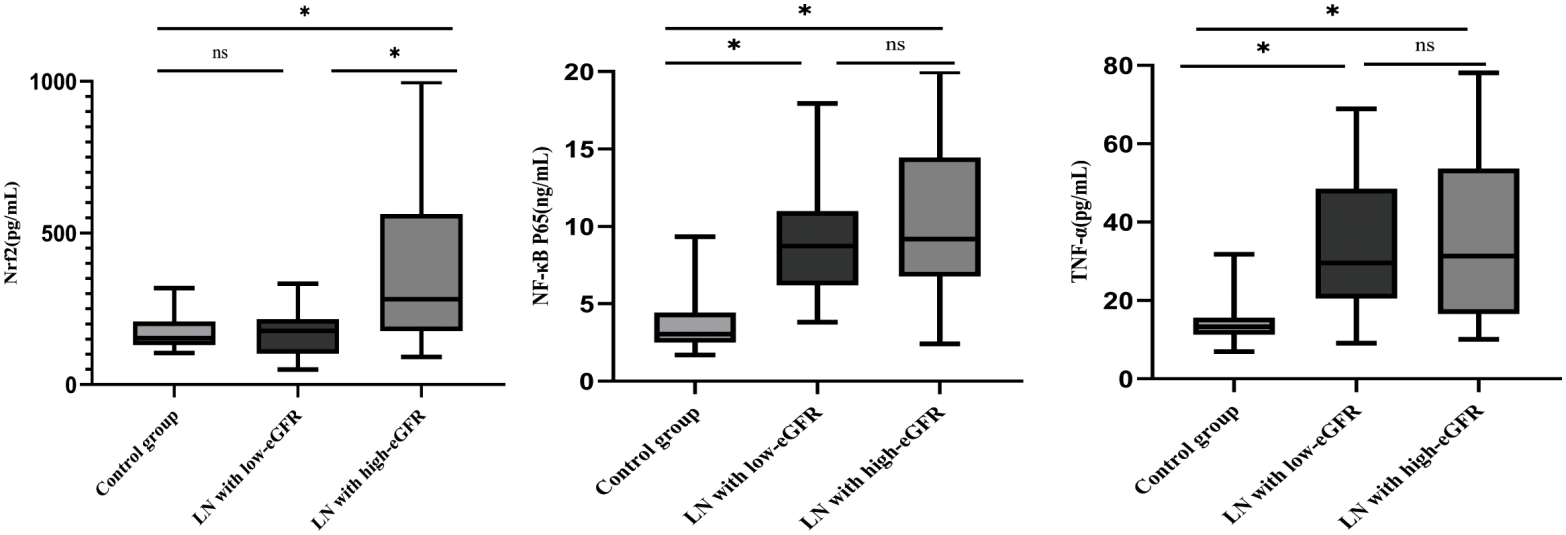
Differences in Nrf2, NF-κB p65, and TNF-α protein levels. Nrf2, NF-κB p65, TNF-α protein concentrations in healthy control subjects, LN with high-eGFR and LN with low-eGFR groups. Serum Nrf2, NF-κB p65, and TNF-α protein concentrations were determined by ELISA. ∗*p* < 0.05. Values of *p* ≥0.05 were deemed to be non-significant.

The differences in Nrf2, TNF-α, and NF-κB p65 protein levels between the three groups are displayed in **Figure 1**. Although the differences in Nrf2 protein levels between the healthy control and LN with low-eGFR groups were not statistically significant, both groups had significantly lower Nrf2 levels than those in the LN with high-eGFR group. The differences between the three groups were: control group vs. LN with high-eGFR group vs. LN with low-eGFR group (177.86 ± 59.43 vs. 387.65 ± 256.07 vs. 165.79 ± 70.11, respectively). After adjusting for age and proteinuria between the LN with low-eGFR and LN with high-eGFR groups using binary logistic regression, there was still a significant difference in Nrf2 protein levels between the two groups (*p* < 0.05), (**Table 4**, **Figure 1**). In addition, the difference in TNF-α protein levels between the LN with high-eGFR and LN with low-eGFR groups was not statistically significant. However, both groups displayed TNF-α protein levels that were significantly higher than that in the healthy control group. The differences between the three groups, namely the control group vs. LN with high-eGFR group vs. LN with low-eGFR group (14.49 ± 5.18 vs. 37.04± 22.26 vs. 33.31 ± 16.42, respectively), and the difference in NF-κB p65 protein levels between the LN with high-eGFR and LN with low-eGFR groups was not statistically significant. Nevertheless, both groups displayed NF-κB p65 protein levels that were significantly higher compared to the healthy control group, and among the three groups: control group, namely LN with high-eGFR group vs. LN with low-eGFR group (3.75 ± 1.98 vs.10.38 ± 5.21 vs.9.06 ± 3.37, respectively).

**Table 4:** Binary logistic regression corrects for age and proteinuria to study serum Nrf2 levels between two groups of LN.

Parameters	B	OR [95% CI]	*P*
Age (years)	0.069	1.071 (1.022,1.122)	0.004^*^
Nrf2(pg/mL)	-0.014	0.987 (0.979,0.994)	<0.001^*^
Proteinuria (g/24 h)	0.426	1.531 (1.166,2.011)	0.002^*^

The analysis was performed with subgroups as (the low-eGFR group - 1, the high-eGFR group - 0) dependent variables. *p<0.05.

### 3.5 Nrf2, TNF-α, and NF-κB p65 protein levels in renal tissues

**Figure 2:**
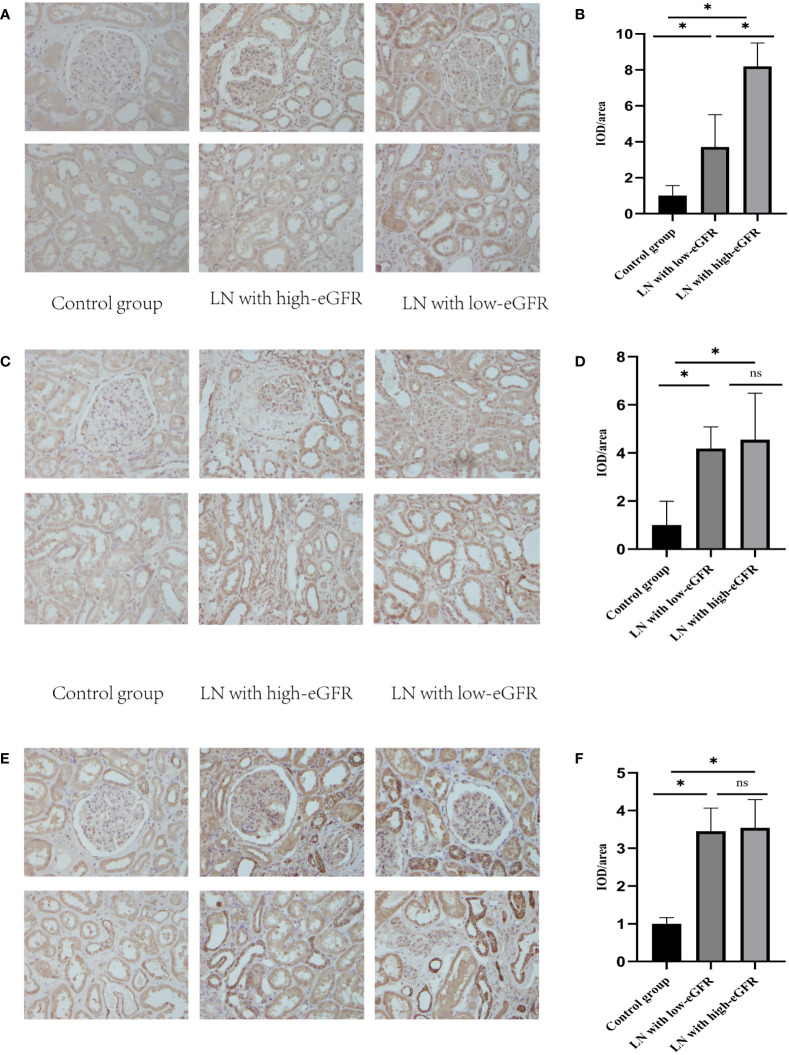
The levels of Nrf2, TNF-α, and NF-κB p65 proteins in renal tissues. **(A, C, E)** are representative micrographs of immunohistochemical staining of Nrf2, NF-κB p65, and TNF-α proteins in renal tissues, respectively. **(B, D, F)** are semi-quantitative estimations of Nrf2, NF-κB p65, and TNF-α protein expression levels, respectively, from the micrographs. Horizontal lines represent mean ± SD. ∗*p* < 0.05.

We assessed the levels of Nrf2, TNF-α, and NF-κB p65 proteins in renal tissues, as shown in **Figure 2**. These data showed that Nrf2 proteins could be expressed in the glomeruli and tubular mesangium, and it was observed that the expression was higher in the tubular mesangium. Nrf2 protein expression levels were significantly higher in both the LN with low-eGFR and LN with high-eGFR groups compared to the healthy controls, whereas they were lower in the LN with low-eGFR group than in the LN with high-eGFR group. After adjusting for age and proteinuria between the LN with low-eGFR and LN with high-eGFR groups using binary logistic regression, there was still a significant difference in Nrf2 protein levels between the two groups (*p* < 0.05), (**Table 5**, **Figure 2A**). Regarding the inflammatory indicators TNF-α and NF-κB p65 detected in renal tissues, the expression levels in both the LN with high-eGFR and LN with low-eGFR groups were significantly higher than those in the healthy control group, but the differences between the two groups were not statistically significant.

**Table 5:** Binary logistic regression corrected for age and proteinuria to study the level of Nrf2 in renal tissue between the two groups of LN.

Parameters	B	OR [95% CI]	*P*
Age (years)	0.057	1.059 (0.973,1.152)	0.183
Nrf2(pg/mL)	-2.388	0.092 (0.024, 0.356)	<0.001^*^
Proteinuria (g/24 h)	0.205	1.228 (0.792,1.903)	0.359

The analysis was performed with subgroups as (the low-eGFR group - 1, the high-eGFR group - 0) dependent variables. *p<0.05.

### 3.6 Effect of the degree of disease activity (SLEDAI score) on Nrf2 protein expression

**Figure 3:**
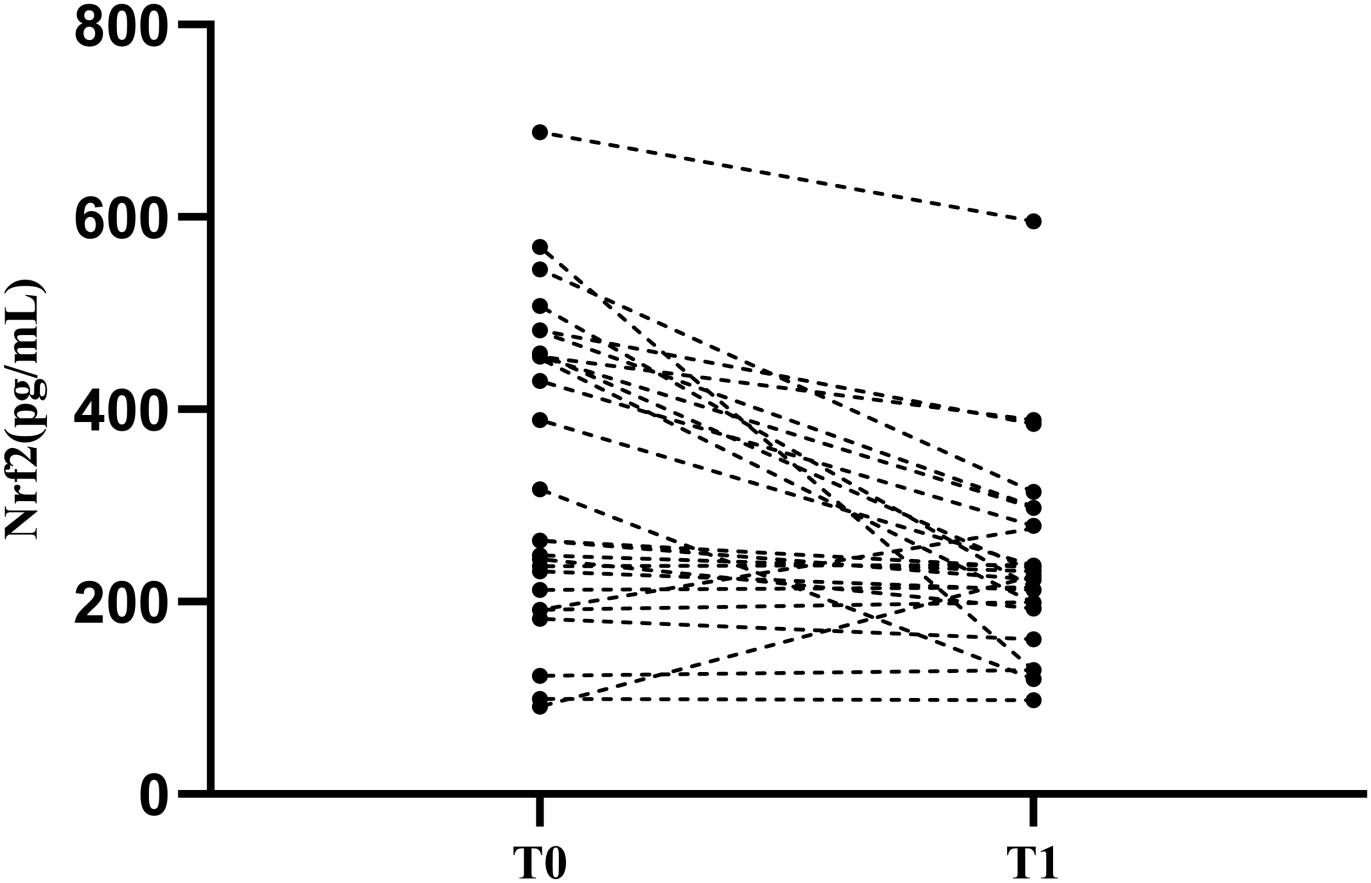
Nrf2 protein levels in patients with LN after treatment. T0: Renal puncture biopsy time point. T1:time point of decreasing disease activity. Patients were observed to have a significant decrease in Nrf2 protein levels after treatment.

Based on these results, we found that Nrf2 protein level decreased significantly after decreases in eGFR. At the same time, Nrf2 protein level correlated with the LN activity level SLEDAI. To verify the effect of disease activity SLEDAI on Nrf2, we followed up the patients in the LN with high-eGFR group. Within the course of 6-12 months follow-up after treatment, disease activity was alleviated (SLEDAI decreased) without observing significant decrease in eGFR. A total of 26 patients were followed up and serum Nrf2 protein levels were measured again (**Figure 3**). We used linear mixed modeling, considering the random effect of time, and included the variables of sex, age, eGFR, and SLEDAI, where sex, age, and eGFR were the control variables. The results showed that SLEDAI correlated with and significantly different from the protein level of Nrf2, with SLEDAI level decreases, Nrf2 protein level decreases as well, **Table 6**.

**Table 6:** Linear mixed modeling of Nrf2 protein levels at baseline and follow-up.

Parameters	OR	95% CI	*P*
Age (years)	0.245	-3.004~3.493	0.880
Sex(male)	-25.415	-142.894~92.064	0.665
eGFR (mL/min/1.73 m^2^)	0.706	-3.597~5.009	0.743
SLEDAI	9.040	0.589~17.491	0.037^*^

*p<0.05.

## 4 Discussion

LN is an important factor in the disability and mortality in patients with SLE (16). Although LN outcome has significantly improved with the use of immunosuppressive and biologic agents, some patients still fail to respond to treatment and develop ESRD (17). Immune complexes deposited during the course of LN trigger a cascading inflammatory response with massive ROS release, which plays a key role in acute and chronic renal lesions in patients with LN (3, 22, 23). Nrf2 is a major regulator of anti-oxidative stress and anti-inflammation, and previous studies have demonstrated that renal injury is more severe in Nrf2-/- mice than in Nrf2+/+ mice in lupus induced using pristane mice (24). An Nrf2-/- genotype exacerbated kidney injury in lupus mice by regulating Th17 cells (25). The level of Nrf2 expression in MRL/lpr mice decreased with the deterioration in LN (26), and Nrf2 ameliorated renal injury in lupus mice by inhibiting ROS and NF-κB (24), indicating that the protective role of Nrf2 in LN has been evident in preclinical studies. In clinical studies, polymorphisms in the human NRF2 gene were associated with pediatric LN (27), and Nrf2 expression was upregulated in the kidneys of patients with LN (24). However, Nrf2 levels decreased in dendritic cells of patients with SLE (28), whereas Nrf2 expression increased in activated T and B cells (21, 29). Accumulating reports support the regulatory role of Nrf2 in LN. Therefore, targeting Nrf2 for LN may be an effective therapeutic option. In preclinical studies, Nrf2 activators such as sulforaphane, dimethyl fumarate, and bardoxolone methyl attenuated inflammation and oxidative stress in lupus mice, as well as it reduced proteinuria and protected renal function (4). Fumarate can treat discoid lupus erythematosus (4). Bardoxolone methyl use has been partially supported in studies on the treatment of type 2 diabetic nephropathy (10). Additionally, Nrf2-mediated oxidative stress pathway is shared in LN and diabetic nephropathy disorders (4). Although these drugs are clinically limited by their therapeutic purposes, their potential for targeting Nrf2 to treat LN still can be promising. Thus, the Nrf2 protein expression levels and precise functions in different immune cell types and tissues remain to be determined. These clinical studies have not yet reflected impaired renal function in patients, thereby do not reflect the state of renal function in patients with LN.

Deficiencies in redox clearance have been reported in patients with SLE. Patients with active SLE have impaired serum and salivary activities of the two oxidative wound-regulating enzymes superoxide dismutase (SOD) and glutathione peroxidase (GPX) (30, 31). Studies have pointed to the recommendation for considering the redox status assessment as a serological marker for assessing disease activity in patients with SLE and renal injury (23). The optimal monitoring strategy for Nrf2 and development of Nrf2 activators have not yet been formulated (32). The expression of Nrf2 protein varies in different immune cell types and tissues of patients with LN (4). This on the one hand may be due to the fact that LN is an autoimmune inflammatory disease; each type of immune cell or tissues may have its own immune response mechanism (with differing impacts on Nrf2 regulation). On the other hand, systemic inflammatory disease is an autoimmune inflammatory disease with varying systemic inflammatory responses, uremic toxins, or comorbidities affecting all cells. It has been shown that Nrf2 levels in patients with chronic kidney disease (CKD) are influenced by inflammation, comorbidities, and the degree of renal damage (33). Therefore, serum Nrf2 protein levels may be used as an assessment for Nrf2 levels in patients with LN.

Our study demonstrated a dual expression of serum Nrf2 protein levels in patients with LN. Nrf2 protein levels in serum and renal tissues were significantly higher in patients with LN than in healthy controls in the absence of decreases in eGFR, when patients may be in a state of high oxidative stress, triggering Nrf2 accumulation with adjacent translocation to the nucleus and induction of antioxidant target genes, thereby activating the Nrf2 system to counteract oxidative stress (34). Our study also indicated that as eGFR decreased to less than 90 ml/min/m^2^, the corresponding Nrf2 protein level decreased compared to normal eGFR. This is supported by the finding that whole blood Nrf2 levels in patients with SLE were lower than those in plasma of healthy controls and patients with CKD (9, 35–37). The decrease in Nrf2 protein levels may be due to renal impairment (due to increased levels of the uremic toxin indoxyl sulfate) limiting the expression of the Nrf2 gene and its protein levels (38, 39). We found that when eGFR was less than 30 ml/min/m^2^, Nrf2 protein levels may be significantly lower compared to healthy controls, possibly due to impaired activation of the Nrf2 system, resulting in an impaired scavenging of ROS (35). In patients with LN, the difference in Nrf2 protein levels between the healthy control group and the low-eGFR group in this study was not statistically significant. This may be related to the different periods of renal injury in which the patients were admitted to the hospital and whether the Nrf2 protein levels were significantly lower than those in the healthy control group. With the increase in the number of patients with severe renal injury that were included, the need for clinical data verification also increased. There is also evidence that prolonged, high levels of Nrf2 activation may have deleterious effects, e.g., in the growth of cancer cells (40), and therefore, further observational studies are warranted on the high levels of Nrf2 in LN under high stress.

Meanwhile, we found that Nrf2 protein expression levels in kidney tissues of patients with LN were significantly higher than that of healthy controls; this is consistent with the results of up-regulation of Nrf2 expression in the kidneys of patients with LN. These data suggest that the kidneys of patients with LN also experience oxidative stress and the body counteracts oxidative stress by eliciting Nrf2 (24). However, we did not determine the extent of kidney injury in these patients. We reported a decrease in Nrf2 expression in renal tissues as eGFR declined, possibly due to the inability of the uremic toxin indoxyl sulfate to be excreted by the kidneys during renal injury, as it accumulates in the body limiting the expression of Nrf2 gene and protein levels. However, unlike serum Nrf2 protein levels, Nrf2 protein in renal tissues of patients with LN in the eGFR-decreased group was still higher than that of healthy controls. This may be because the serum Nrf2 protein levels could be more sensitive to stimuli than did the renal tissue. It should also be taken into account that the number of patients enrolled in our IHC experiment might be insufficient or its expression is affected by the type of pathology (24). NF-κB and TNF-α are involved in the progression of LN (41, 42), and we simultaneously investigated the inflammatory indices, NF-κB p65 and TNF-α levels, in serum and renal tissues of patients with LN, and found that NF-κB p65 and TNF-α expression in LN was significantly higher than that in healthy controls. There was no statistically significant difference in the expression of NF-κB p65 and TNF-α in the groups with different eGFRs, and this indicated that inflammation still existed in the group with low-eGFR, whereas the Nrf2 protein levels decreased significantly in this group, probably because Nrf2 expression was affected by the uremic toxin indoxyl sulphate concentration: this highlights the dual role of Nrf2 in LN.

In the present study, Nrf2 protein levels were positively correlated with disease activity (SLEDAI scores), and previous studies have also pointed out that Nrf2 expression in B cells of patients with SLE positively correlated with disease severity (SLEDAI score) (21). We further verified the relationship between SLEDAI scores and Nrf2 protein expression by following up with similar patients with who did not have a significant decrease in eGFR. The significant decrease in Nrf2 protein levels in patients treated with induction therapy reinforces the fact that patients are in a state of high oxidative stress during the active phase of the disease that triggers Nrf2 expression aimed at responding to antioxidants. When relieved by induction therapy, the level of Nrf2 protein decreases, and this may be due to the alleviation of oxidative stress *in vivo* (24).

This study has some limitations: First, the relatively low patient numbers included in this study were insufficient for analyzing each stage of renal injury, and thus the statistical results need to be interpreted with more caution. We hope that in the future, Nrf2 protein concentrations will be analyzed independently in patients with LN at each stage and in different disease states. At the same time, other indicators of oxidative stress and Nrf2 activity were not tested in this experiment to assess the level of oxidative stress *in vivo* and determine the relationship between Nrf2 activity and disease, which will need to be assessed in more future studies.

In conclusion, our study found that Nrf2 protein levels were significantly higher in patients with LN when they displayed a normal glomerular filtration rate (eGFR ≥90 ml/min/m^2^) than when they had reduced glomerular filtration rates (eGFR <90 ml/min/m^2^) and decreased when LN was in remission. Therefore, the detection of serum Nrf2 protein levels could be a promising strategy for replacing its detection in various cells or tissues as a predictor of disease activity and renal impairment in patients with LN. Nrf2 protein levels should be evaluated and correlated with the disease state and the extent of renal impairment in patients with LN prior to targeted Nrf2 therapy. This approach would provide theoretical support for the development and individualization of Nrf2 targeted therapy in clinical practice.

## Data availability statement

The raw data supporting the conclusions of this article will be made available by the authors, without undue reservation.

## Ethics statement

The studies involving humans were approved by the Human Investigation Committee of the Second Hospital of Jilin University. The studies were conducted in accordance with the local legislation and institutional requirements. The participants provided their written informed consent to participate in this study. Written informed consent was obtained from the individual(s) for the publication of any potentially identifiable images or data included in this article.

## Author contributions

JL: Writing – original draft. QG: Validation, Funding acquisition, Writing – original draft. XW: Data curation, Writing – original draft. YZ: Methodology, Writing – original draft. ML: Data curation, Funding acquisition, Writing – review & editing. PL: Funding acquisition, Writing – review & editing.
