# Supplementary material for: Association of serum Nrf2 protein levels with disease activity and renal impairment in lupus nephritis
Source: Front Immunol. 2024 Jan 18;15:1304167. doi: 10.3389/fimmu.2024.1304167 (PMC10830626; doi:10.3389/fimmu.2024.1304167)
Supplement: Supplementary file 1 [file Table_1.docx]

| **Entry criterion**  Antinuclear antibodies (ANA) at a titer of ≥1:80 on HEp-2 cells or an equivalent positive test (ever). | | | |
| --- | --- | --- | --- |
| If absent, do not classify as SLE  If present, apply additive criteria | | | |
| **Additive criteria**  **Do not count a criterion if there is a more likely explanation than SLE.**  **Occurrence of a criterion on at least one occasion is sufficient.**  **SLE classification requires at least one clinical criterion and ≥10 points.**  **Criteria need not occur simultaneously.**  **Within each domain, only the highest weighted criterion is counted toward the total scores.** | | | |
| **Clinical domains and criteria** | **Weight** | **Immunology domains and criteria** | **Weight** |
| **Constitutional**  Fever | 2 | **Antiphospholipid antibodies**  Anti-cardiolipin antibodies OR  Anti-β2GP1 antibodies OR  Lupus anticoagulant | 2 |
| **Hematologic**  Leukopenia  Thrombocytopenia  Autoimmune hemolysis | 3  4  4 | **Complement proteins**  Low C3 OR low C4  Low C3 AND low C4 | 3  4 |
| **Neuropsychiatric**  Delirium  Psychosis  Seizure | 2  3  5 | **SLE-specific antibodies**  Anti-dsDNA antibody*OR  Anti-Smith antibody | 6 |
| **Mucocutaneous**  Non-scarring alopecia  Oral ulcers  Subacute cutaneous OR discoid lupus  Acute cutaneous lupus | 2  2  4  6 |  |  |
| **Serosal**  Pleural or pericardial effusion  Acute pericarditis | 5  6 |  |  |
| **Musculoskeletal**  Joint involvement | 6 |  |  |
| **Renal**  Proteinuria>0.5g/24h  Renal biopsy Class II or V lupus nephritis  Renal biopsy Class III or IV lupus nephritis | 4  8  10 |  |  |
| **Total score: Classify as Systemic Lupus Erythematosus with a score of 10 or more if entry criterion fulfilled.** | | | |

**2019 EULAR/ACR Classification Criteria for Systemic Lupus Erythematosus**
